# X-ray focusing with efficient high-NA multilayer Laue lenses

**DOI:** 10.1038/lsa.2017.162

**Published:** 2018-03-23

**Authors:** Saša Bajt, Mauro Prasciolu, Holger Fleckenstein, Martin Domaracký, Henry N Chapman, Andrew J Morgan, Oleksandr Yefanov, Marc Messerschmidt, Yang Du, Kevin T Murray, Valerio Mariani, Manuela Kuhn, Steven Aplin, Kanupriya Pande, Pablo Villanueva-Perez, Karolina Stachnik, Joe PJ Chen, Andrzej Andrejczuk, Alke Meents, Anja Burkhardt, David Pennicard, Xiaojing Huang, Hanfei Yan, Evgeny Nazaretski, Yong S Chu, Christian E Hamm

**Affiliations:** 1 Photon Science, DESY, Notkestrasse 85, Hamburg 22607, Germany; 2 Center for Free-Electron Laser Science, DESY, Notkestrasse 85, Hamburg 22607, Germany; 3 Department of Physics, University of Hamburg, Luruper Chaussee 149, Hamburg 22607, Germany; 4 Centre for Ultrafast Imaging, Luruper Chaussee 149, Hamburg 22607, Germany; 5 National Science Foundation BioXFEL Science and Technology Center, 700 Ellicott Street, Buffalo, NY 14203, USA; 6 Department of Physics, Arizona State University, Tempe, AZ 85287, USA; 7 Faculty of Physics, University of Bialystok, Ciolkowskiego 1L Str., Bialystok 15-245, Poland; 8 National Synchrotron Light Source II, Brookhaven National Laboratory, Upton, NY 11973, USA; 9 Alfred-Wegener Institute, Helmholtz Center for Polar and Marine Research, Bussestr. 27, Bremerhaven 27570, Germany

**Keywords:** multilayer Laue lenses, multilayers, ptychography, X-ray holography, X-ray optics

## Abstract

Multilayer Laue lenses are volume diffraction elements for the efficient focusing of X-rays. With a new manufacturing technique that we introduced, it is possible to fabricate lenses of sufficiently high numerical aperture (NA) to achieve focal spot sizes below 10 nm. The alternating layers of the materials that form the lens must span a broad range of thicknesses on the nanometer scale to achieve the necessary range of X-ray deflection angles required to achieve a high NA. This poses a challenge to both the accuracy of the deposition process and the control of the materials properties, which often vary with layer thickness. We introduced a new pair of materials—tungsten carbide and silicon carbide—to prepare layered structures with smooth and sharp interfaces and with no material phase transitions that hampered the manufacture of previous lenses. Using a pair of multilayer Laue lenses (MLLs) fabricated from this system, we achieved a two-dimensional focus of 8.4 × 6.8 nm^2^ at a photon energy of 16.3 keV with high diffraction efficiency and demonstrated scanning-based imaging of samples with a resolution well below 10 nm. The high NA also allowed projection holographic imaging with strong phase contrast over a large range of magnifications. An error analysis indicates the possibility of achieving 1 nm focusing.

## Introduction

X-ray-based techniques enable non-destructive measurements of natural and man-made materials and provide elemental, chemical and structural information of the internal structures of these materials. These techniques range from diffraction of crystalline materials at the atomic scale to tomographic imaging of large organisms such as humans. Understanding the structure and function of materials on the nanometer scale allows modifications and custom designs of new materials with improved properties, with applications in fields spanning medicine, health, energy, environment, electronics and transport. Recent advances in bright, coherent, and laser-like X-ray sources give the potential to extend imaging to high resolution without necessary restrictions on the sample crystallinity or high degrees of order. Combined with high-efficiency high-numerical-aperture (NA) lenses, such developments might eventually enable investigation of non-crystalline and disordered matter at the scale of molecules—that is, at length scales of approximately 1 nm.

**Figure 1:**
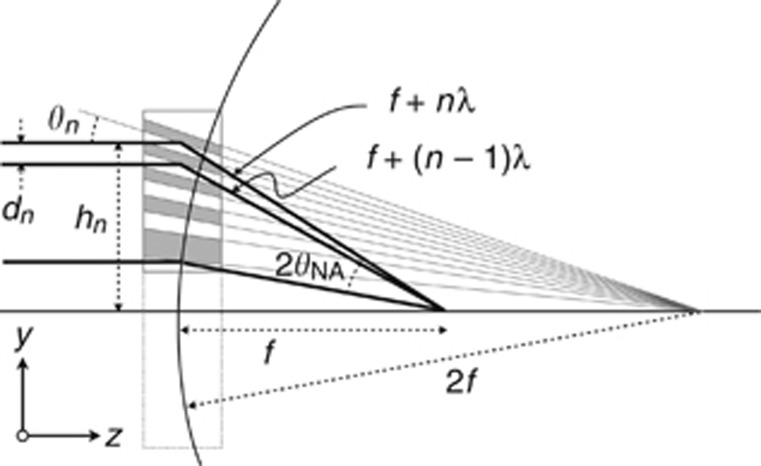
To achieve high diffraction efficiency across the entire pupil of a multilayer Laue lens, the layer periods *d*_*n*_ at heights *h*_*n*_ must follow the zone plate law such that reflected rays constructively interfere at the focus. The layers must be wedged so that Bragg’s law sin *θ*_*n*_=*λ*/(2*d*_*n*_) is satisfied locally at every bi-layer for a wavelength λ. For a lens of focal length *f,* this places the layers normal to a circle of radius 2*f*. The lens can be thought of as an off-axis portion of a larger parent lens. The numerical aperture is given by sin *θ*_*NA*_, where *θ*_*NA*_ is half of the difference between the largest and smallest deflection angles.

Although there are different ways to focus X-rays^1^, the most promising way to achieve a nanometer-resolution X-ray microscope is with a volume zone plate^2, 3^. This diffractive optical element consists of alternating layers of two materials of differing densities, with layer periods that decrease as the number of bi-layers *n* increases from the optic axis following the Fresnel zone-plate formula given approximately by 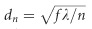 for a focal length *f* and wavelength λ (see Figure 1). This arrangement ensures that layers (or zones) further from the optic axis diffract rays by steeper angles such that they all converge at a focal point. For any lens, the spot size, or resolution, of the lens is governed by the range of deflection angles of rays converging on that point. The largest deflection angle is set by the smallest layer period; thus, layer periods below 1 nm are required with X-ray wavelengths that are considerably shorter than this. To achieve a lens with a high efficiency, the lens structure must be as thick as the extinction depth for diffraction, which is several micrometers for such wavelengths. If the NA is larger than the inherent width of the diffraction rocking curve of the multilayer structure, then only a portion of the lens will reflect rays, effectively reducing it to a smaller aperture with worse resolution. To utilize the entire aperture of the lens, each layer in the lens must be at the proper angle to ensure that Bragg’s law is satisfied for every period in the structure. This means that the orientation of the layers must vary throughout the layer stack, giving rise to a wedged structure, as shown in Figure 1. The diffraction properties are described by dynamical diffraction theory, which predicts oscillations of intensity shared between just the zero and first (focusing) orders with optical depth, with almost 100% diffraction efficiency possible^4, 5^.

For soft X-ray wavelengths, the strong interaction with materials ensures that efficient diffraction can be achieved with thin structures—Fresnel zone plates—which can be manufactured by electron beam lithography. This approach is not appropriate for the structures mentioned above, with nanometer zone widths of several micrometers thickness. Structures with such large aspect ratios can be easily obtained instead by the deposition of two or more materials onto a substrate using magnetron sputtering and then slicing this structure to the desired thickness. Early attempts to create structures from cross-sections of multilayer films, as in ‘sputter/slice’ zone plates deposited on wires, demonstrated that very high aspect ratios could be produced. These studies also revealed challenges, such as achieving uninterrupted deposition of thousands of layers, attaining the necessary accuracy in the placement of those layers, and controlling their stress and maintaining their structure during sectioning^6, 7, 8, 9^. The state of the art of this approach reached 5 nm resolution by depositing a multilayer with a thickness of 350 nm onto a 900 nm diameter wire^10^. An important step forward was the demonstration of Laue diffraction (in which the reflected beam exits a different face of the structure than the incident beam) by sectioned multilayers made on flat substrates, giving highly efficient diffraction in agreement with the dynamical theory^11^. Two such lenses, in a crossed geometry, are needed for two-dimensional focusing, similar to two crossed cylindrical lenses^12^. This development was a result of major advancements in deposition technology related to the semiconductor industry’s interest in extreme ultraviolet lithography. This began the development of a new generation of hard X-ray optics, referred to as MLLs^13^.

The first demonstration of one-dimensional (1D) focusing with a MLL constructed from parallel layers resulted in a 16 nm focal spot at a photon energy of 20 keV^14^. The NA of that lens was limited by the acceptance angles, or the width of the rocking curve, of the parallel layers, as mentioned above. An MLL with 6510 parallel layers and a 4 nm outermost layer period produced a near-diffraction limited focus of 11 nm with a 4.2 mm focal length at 12 keV^15^. Recently, a higher-NA MLL was demonstrated with a 1D focus of 8 nm at 22 keV^16^. The NA of 0.006 was achieved by constructing the lens with wedged layers to obtain useable diffraction efficiency throughout the entire height of the lens. That lens consisted of tungsten (W) and silicon carbide (SiC) layers, and the wedging of the layers was achieved within the penumbra of the deposition particles cast by a straight mask placed above the substrate^17, 18^. This method permits the required control of the grading of the layers: optimization for a particular photon energy and focal length can be performed by choosing the location in the penumbra where the MLL is cut. For 2D focusing, two MLLs are required, each optimized for the same energy but with different focal lengths.

**Figure 2:**
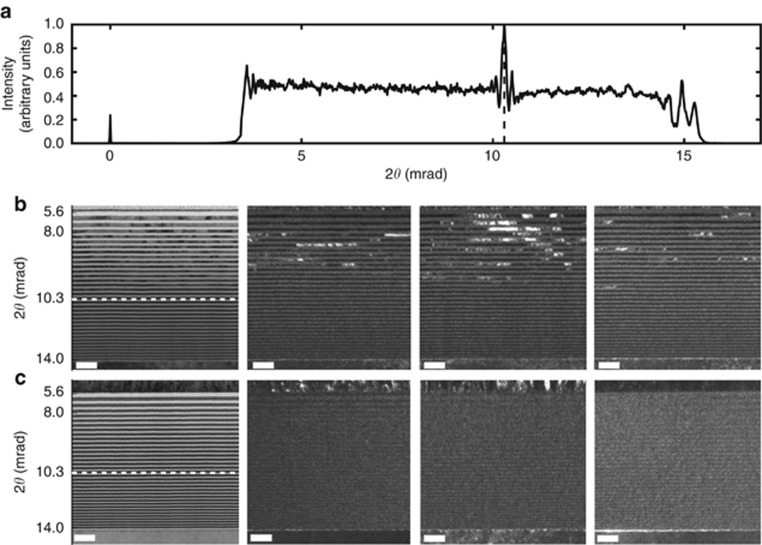
Plot of the far-field diffracted intensity as a function of the angular position from the W/SiC MLL (**a**) (adapted from Ref. 16). A localized phase error at a scattering angle of approximately 10.3 mrad at 0.056 nm wavelength from the W/SiC lens gave rise to an obvious intensity spike^16^. At that position, the multilayer period was approximately 5.5 nm. Bright field (left column) and dark field (remaining columns) TEM images of W/SiC (**b**) and WC/SiC (**c**) have periods from 4.0 to 10 nm (as described in the Materials and Methods section). The white bar in all images corresponds to 20 nm. The transition from amorphous to crystalline W layers occurs at a period of approximately 5.7 nm.

Because the resolution of an MLL is dependent upon its smallest period, it is essential to use a multilayer that forms sharp and stable interfaces. Furthermore, a high-quality lens requires low film stress, low interface roughness, and no defects or interruptions in the multilayer structure. Tungsten/silicon carbide (W/SiC) multilayers form sharp and stable interfaces even for periods as small as 1 nm, as needed for applications well above 100 keV^19, 20, 21^, making them an attractive choice for MLLs. However, when we fabricated MLLs from these materials and characterized them by ptychography, we discovered that the pupil wavefront contained a discontinuity that gave rise to a pronounced modulation of the far-field X-ray intensity profile of the lens, as shown in Figure 2 (Ref. 16). Here, we trace the cause of this discontinuity to a transition of the microstructure of the W layers from an amorphous state to a crystalline state that occurs abruptly as the layer thickness exceeds 3 nm. Such effects have been observed in Mo-based multilayer systems^22^ and are known to affect the intrinsic stress^23^. The discontinuity resulted in two separate foci^16^, each formed from a different region of the lens. Since each region is smaller than the entire lens, they separately produce larger foci and thus a significant decrease in resolution compared with a lens without such aberrations. While this discontinuity could be avoided by designing the lens to operate further from the optic axis (with the entire range of layer thicknesses smaller than the transition thickness of 3 nm), we sought new materials where such effects are absent.

We found the requisite multilayer structure by replacing metallic W with its carbide, WC. We present a characterization of high NA MLLs fabricated using this new material pair and find that these materials possess the desired characteristics of smoothness and regularity while remaining amorphous. We created a set of two lenses with 0.0075 NA for 2D imaging at 16 keV (0.077 nm wavelength) by employing fabrication methods described in the Materials and Methods section. The lenses were characterized by ptychography and scanning transmission imaging (Results and Discussion section), with an analysis of the aberrations indicating a path towards X-ray imaging at 1 nm resolution.

## Materials and methods

All multilayers employed in this study were deposited in DESY’s multilayer X-ray lab using magnetron sputtering. More details about the deposition process and MLL fabrication are presented elsewhere^17, 18^. The DC sputtering power was 220 W for the SiC target, 100 W for W, and 100 W for WC, which produced deposition rates of 0.3 nm s^−1^ for SiC, 0.65 nm s^−1^ for W and 0.6 nm s^−1^ for WC.

To investigate the dependence of the microstructure of the layer materials as a function of their thickness, W/SiC and WC/SiC multilayers were prepared in which the layer thickness varied throughout the stack. Each multilayer stack consisted of 34 layer pairs, beginning with a period of 4.0 nm, which was increased to 7.0 nm in 0.1 nm steps. From that point, the layers were increased in period from 7.0 to 10.0 nm in 1 nm steps. The two layer materials were deposited with equal thickness ratios for all periods. These ‘depth graded’ multilayers are, of course, unsuitable for focusing.

The two MLLs fabricated for this study were designed to focus 16 keV X-rays with 0.0075 NA and focal lengths of 1.4 and 2 mm to create a 2D focus when placed 0.6 mm apart. The Rayleigh resolution for an ideal lens pair with this geometry is 5.0 nm in the horizontal and vertical directions (for a lens with a square pupil, the Rayleigh resolution is given by 0.5 *λ*/NA). Each multilayer structure was deposited on a super-polished Si wafer with a roughness of <0.2 nm rms at spatial frequencies assessable through AFM measurements. The lens parameters are given in Table 1. Both lenses were designed as off-axis sections of an ideal parent lens. This partial structure avoids the need to deposit layers thicker than approximately 30 nm, which would otherwise lead to additional stress and roughness, and gives the advantage that the direct (zero order) and deflected beams are well separated at the focal plane. To take full advantage of the smooth substrate, we started the deposition with the thinnest layers. Each lens was cut from the multilayer film using a focused ion beam (FIB) in an FEI Helios Nanolab Dual Beam FIB/SEM with a thickness of 6.5 μm in the direction of the optic axis, calculated as giving optimum diffraction efficiency for 16 keV^5^. Each MLL was transferred to a separate mount, which enables the independent positioning and alignment of each lens.

The two depth-graded multilayers were investigated in cross section using transmission electron microscopy (TEM). Their post-deposition preparation involved using an *in situ* focused ion beam (FIB) lift-out technique on an FEI Dual Beam FIB/SEM (Evans Analytical Group, USA). TEM images were obtained with an FEI Tecnai TF-20 FEG/TEM operated at 200 kV in bright-field (BF), dark-field (DF), and a high-resolution TEM (HRTEM) mode.

**Figure 3:**
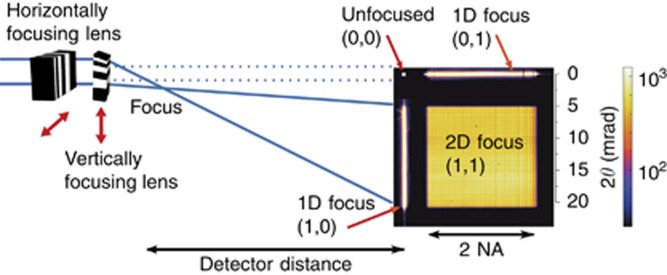
Experimental setup used at the P11 and HXN beamlines. Two MLLs are orthogonal to each other, as indicated by the red double-headed arrows. At P11, a LAMBDA detector with 55-μm pixels was used to measure the far-field intensity at a distance 1.4 m from the focus. At HXN, a Timepix detector with 55-μm pixels was placed 0.533 m downstream of the focus. An example of the efficiency measurement is shown from P11, with intensities shown on a logarithmic color scale.

To characterize the diffraction efficiencies and wavefront aberrations of the MLLs and to form images using the lenses, we used beamlines P11 (Refs. 24, 25) at the PETRA III synchrotron radiation facility (DESY, Hamburg) and the Hard X-ray Nanoprobe (HXN) beamline at the National Synchrotron Light Source II (NSLS-II) of Brookhaven National Laboratory (NY, USA)^26, 27^. At both these facilities, we used a scanning microscopy geometry, whereby a monochromatized collimated beam was focused by the lenses to illuminate a spot on the sample that was scanned in its transverse position. At the HXN beamline, where we used a slightly modified test system to accommodate our MLLs, a portion of the monochromatized beam was selected by an aperture placed in the secondary source of the beamline, approximately 8 m upstream of the lenses, to achieve a spatial coherence length matched to the width of the lenses. Each lens in its mount was attached to an independent and motorized stage with both translational and rotational degrees of freedom with linear and angular resolutions of approximately 0.1 μm and 5 μrad, respectively^28, 29^. The lenses were mounted to focus in the horizontal and vertical directions. The far-field diffractions from the lenses alone and with the sample in place were recorded using a pixel-array detector. At P11, this was a LAMBDA 750 K detector (X-Spectrum) placed 1.4 m downstream from the focus. This had 512 × 1536 pixels with an individual pixel width of 55 μm, which was monitored in real time using the OnDa software package^30^. At HXN, a Timepix detector (512 × 512 pixels, 55 μm width) was placed 0.533 m downstream of the focus. The general scheme is depicted in Figure 3.

Characterization of the lens pair was carried out by scanning a sample in or near the plane of the beam focus and recording the far-field intensity pattern for each sample position. The off-axis focusing geometry of the lenses, apparent in the separation of orders in the far field, as shown in Figure 3, also results in a separation of these orders in the focal plane by the distance of the low-angle edge of the lens to the optic axis. Initial alignment of the MLLs was achieved by bringing each lens into the diffracting condition by observing the extinction of the direct beam on a downstream YAG screen. When the Bragg condition is satisfied, the lens appears black because most of the light is diffracted, while the unaligned lens transmits the X-rays and is therefore transparent (Supplementary Fig. S1). A series of knife-edge scans was made to bring the two foci to the same focal plane. To eliminate 45° astigmatism, the lenses were adjusted to be orthogonal^31^.

## Results and discussion

### Microstructure of the layer materials

To develop high-NA MLLs, it is of utmost importance to understand the microstructure, intrinsic stress, and interface roughness evolution of layers as a function of period thickness. The need for this understanding was apparent from the observation of a localized phase error in our W/SiC MLLs at a scattering angle of 10.3 mrad with 22 keV X-rays, as shown in Figure 2a, corresponding to a period of 5.5 nm^16^. We hypothesized^16^ that the discontinuity was caused by an amorphous-to-crystalline transition in the metallic W layers with their increasing thickness. Crystals usually nucleate in sputter-deposited metal layers thicker than a few nanometers. The two depth-graded samples described in the Materials and Methods section were prepared to investigate this hypothesis and to qualify our new material pair, WC/SiC, to produce structures without the transition (Supplementary Fig. S2).

HRTEM images of these two depth-graded multilayers are presented in Figure 2b (W/SiC) and Figure 2c (WC/SiC). In the bright field images (left column), the contrast arises due to the differences in the material density: the higher-density material (W or WC) is darker than the lower-density material (SiC). The thickest W layers at the top of the W/SiC multilayer contain crystalline material (Figure 2b). No evidence of crystalline material is observed in the WC layers of the WC/SiC multilayer (Figure 2c). In both multilayer systems, the SiC layers appear fully amorphous. The presence of a crystalline material is easier to detect in dark field mode (remaining columns). In dark field imaging, an aperture is placed in the back focal plane of the objective lens of the TEM and displaced away from the optic axis so that the electrons that are not scattered by the sample are blocked. Thus, the image is dark when there is no sample, and a signal is only achieved in places in the field of view where the sample scatters electrons in the direction of the aperture. We set the displacement of the aperture from the optic axis to coincide with the first-order diffraction angle of crystalline W and moved the aperture to different azimuthal positions to visualize crystallites in different orientations. Three such images are shown in Figure 2b and 2c. The transition is taken to occur at the thinnest layer where crystallites were observed in any one or more orientations (bi-layer 18 in Figure 2b). There, the bi-layer is 5.7 nm thick, and the W layer thickness is 2.85 nm. Using selected area electron diffraction patterns, it is possible to identify these crystallites as W with a W-bcc structure. Observing the SiC and WC layers in dark field mode and checking at different angles, such that no such bright areas could be detected, confirmed their amorphous nature.

The thinnest periods containing crystalline material in the HRTEM images of Figure 2 agree with the period of layers occurring at the position of the observed phase error in the W/SiC MLLs. Furthermore, the positions of the two foci determined for those lenses^16^ agree with the fact that the crystalline form of W has higher density than the amorphous form.

### Characterization of diffraction efficiency

**Figure 4:**
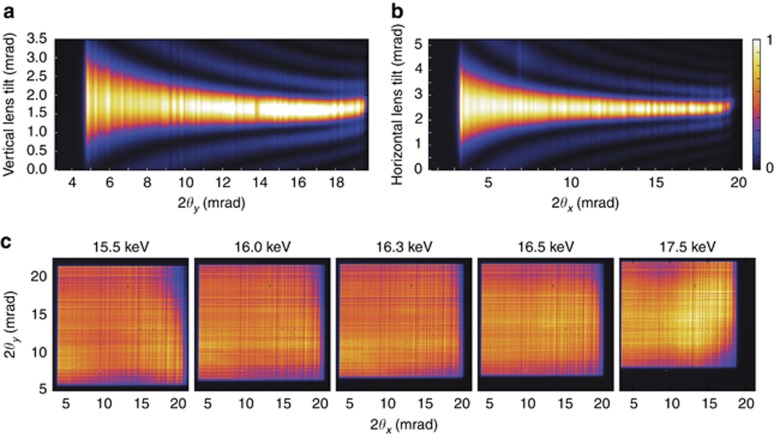
Diffraction efficiency of the (**a**) vertical and (**b**) horizontal lenses, as determined by the far-field 1D diffraction pattern of each lens at 16.3 keV photon energy, mapped as a function of tilt of the MLL lens. (**c**) Maps of the relative diffraction efficiency of the two lenses combined, from the far-field 2D diffraction pattern as a function of photon energy, ranging from 15.5 to 17.5 keV. All intensity maps are shown on a linear color scale. The side of the square pupil corresponds to 20 mrad (at 16.3 keV), and it scales inversely with photon energy.

The diffraction characteristics of the lenses were determined using the scanning microscope setup described in the Materials and Methods section. A series of scans of the tilt of the lens was performed at different photon energies to find the optimum energy and tilt of each individual lens. Each tilt scan was constructed from one-dimensional far-field pupil intensity profiles recorded on the LAMBDA detector as a function of scattering angle, similar to that shown in Figure 2a, and then mapped as a function of the rocking angle of the lens, as shown in Figure 4a and 4b. The correct photon energy was indicated by a map that gave a horizontal trace of maximum intensity since this indicates high diffraction efficiency across the entire pupil of the lens.

A final optimization of lens tilts was subsequently carried out with both lenses in the beam to create a two-dimensional focus. Since each lens diffracts into both the focused order and the zero order, the far-field pattern consists of four distinct regions: the zero-order transmission of both lenses, denoted (0, 0) in Figure 3, the zero order from one lens combined with the focus of the other, (0, 1) and (1, 0), and the two-dimensional focus from both lenses (1, 1). No other orders are observed. To achieve a uniform efficiency across the entire two-dimensional pupil, final adjustments of the tilts of the lenses were performed while monitoring the far-field pattern. It was necessary to adjust the tilts in the orthogonal directions to their Bragg rocking directions since the first lens changes the direction of the rays illuminating the second lens. Examples of the pupil efficiencies at different photon energies for the pair of lenses used in this study are shown in Figure 4c. The design energy was 16 keV, and the best performance for the combination was found at 16.3 keV.

The ability to measure the combinations of diffracted orders, as shown in Figure 3, allows the efficiencies of each individual lens to be determined. We label the horizontally focusing lens as MLL1 and the vertically focusing lens as MLL2. Taking the focusing efficiency (fraction of diffracted intensity in the focusing order) of MLL1 to be *e*_1_ and that of MLL2 to be *e*_2_, the fraction of photons focused by both MLL1 and MLL2 is *s*=*e*_1_ × *e*_2_ (for the square pupil). The fraction of photons diffracted to focus by MLL1 but not MLL2 is *h*=*e*_1_ × (1−*e*_2_); similarly, the fraction of photons diffracted to focus by MLL2 but not MLL1 is *v*=*e*_2_ × (1−*e*_1_). Thus, one can calculate the relative focusing efficiency of the lenses from the ratio of the integrated photon counts in the one-dimensional to the two-dimensional foci:


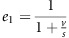



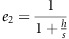


We measure *v*/*s*=0.170 and *h*/*s*=0.244, giving estimated efficiencies of our lenses as 85.5 and 80.4%, accordingly. The relative efficiency for two-dimensional focusing, calculated as the product of individual lens efficiencies, is 68.7%. These estimates do not include any overall absorption in the lenses. This can be estimated by measuring the transmission of the lenses when tilted away from the diffracting condition, which was found to be over 80%, as observed on a downstream YAG screen.

### Focus reconstruction and scanning transmission X-ray microscopy (STXM) images

**Figure 5:**
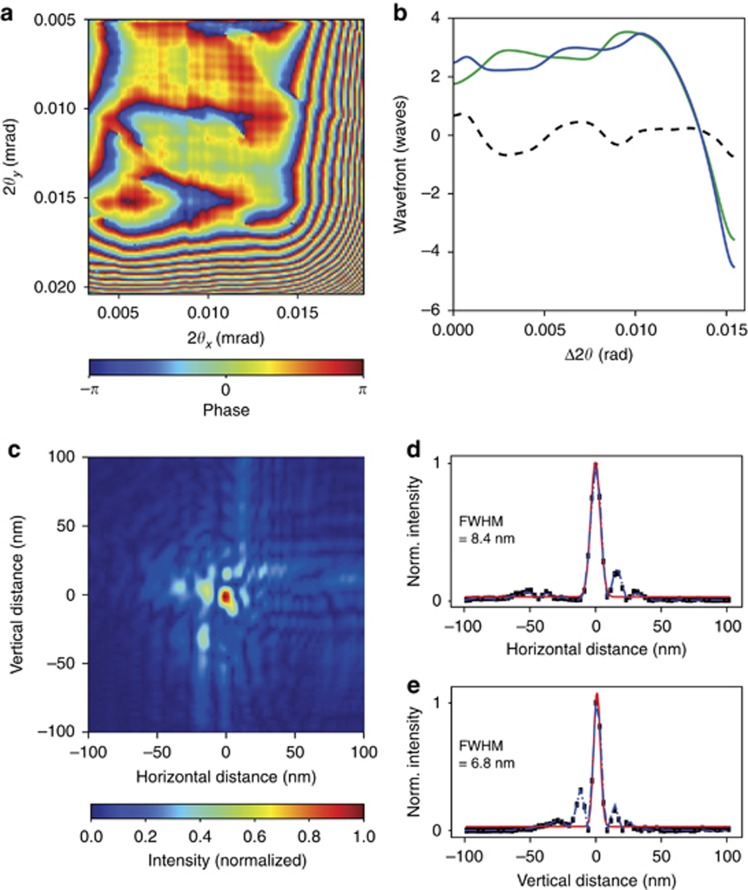
(**a**) Wavefront error in the pupil plane of the MLL showing significant error at the edges of the lenses. (**b**) The unwrapped wavefront separated into 1D phase profiles of each individual lens, and the differences in the phase error of the two lenses (dashed line) indicate an upper limit of the manufacturing reproducibility. (**c**) Reconstructed intensity in the MLL focus as determined by ptychography. Lineouts in horizontal (**d**) and vertical directions (**e**) of the in-focus intensities (black dots) were fitted with Gaussian functions (red lines) with widths of 8.4 and 6.8 nm, respectively. The color bars in (**a**) and (**c**) indicate the normalized phase and intensity.

A gold star-shaped test pattern consisting of radial spokes with the finest feature being 20 nm was scanned across the focused beam at a plane 20 μm downstream of focus at the HXN beamline. A total of 512 diffraction frames were collected over a 1 μm × 1 μm area in a scan following the trajectory of a Fermat spiral^26^. Data arrays cropped to 256 × 256 pixels were reconstructed using 200 iterations of the difference map algorithm^32^ to give the complex-valued transmission function of the object and the wavefront of the probe with 2.9 nm real-space sampling resolution. The probe function can be transformed to the plane of the lens via a Fourier transform to give the complex-valued lens pupil function. The phase term describes the aberrations, which includes a significant quadratic term due to the 20 μm of defocus. Subtracting this term (and piston and tilt) gives the wavefront aberration of the lens, as shown in Figure 5a. It is seen that the wavefront at two neighboring edges of the square pupil have a significant error of 8 waves, peak to valley. These edges of the pupil correspond to the edge of each MLL where the diffraction angle is highest—that is, regions where the layer period is smaller than approximately 5.4 nm.

Since the pupil function is formed by two cylindrical lenses, it is expected that the wavefront aberration *φ*(*x*, *y*) is separable into functions of *x* and *y* (the directions of the two lenses). This is indeed the case, and these functions, *φ*_1_(*x*) and *φ*_2_(*y*), are obtained by integrating the phase in the *x* or *y* direction and then normalizing appropriately. The normalized mean square error |*φ*(*x*, *y*)−*φ*_1_(*x*)*φ*_2_(*y*)|^2^/|*φ*(*x*, *y*)|^2^ is found to be less than 18%. These individual phase functions of lenses 1 and 2 are given in Figure 5b. It is apparent that the errors are very similar, even though the lenses were fabricated in different deposition runs.

Despite the large roll-off of the aberration at the two edges of the pupil, the reconstructed focal spot size is 8.4 × 6.8 nm^2^, as shown in Figure 5c. Line-outs of the focal intensity distribution are shown in Figure 5d and 5e. The Rayleigh width of the focal spot for a square pupil of 0.0075 NA for 16 keV is 5.0 nm. We note that if we were to decrease the pupil size by blocking the high-angle edges of the lenses, then a wavefront error of 0.2 waves RMS could be achieved with an NA of 0.006 after subtracting piston, tilt, and defocus. Lineouts of the profiles that would be obtained in that case are also shown in Figure 5d and 5e, giving focal widths equal to that with the full pupil and showing that the wavefront roll-off at the two edges directs light completely out of the focal spot.

**Figure 6:**
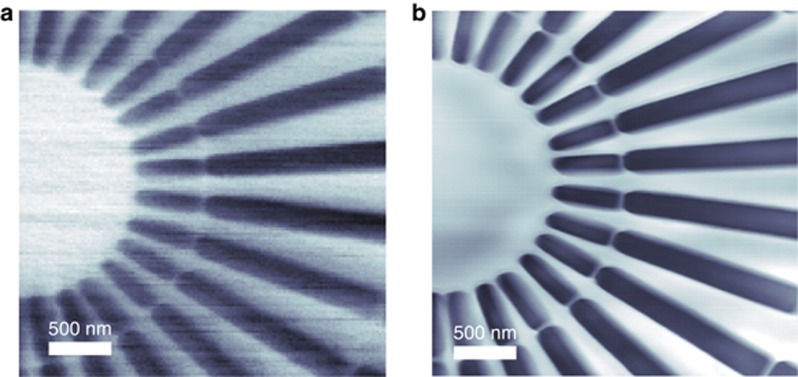
The incoherent STXM absorption-contrast image (**a**) and ptychography reconstructed image (**b**) showing a Siemens star with 100 nm inner spikes and a 20 nm spacing ring between the inner and outer spikes.

A second scan was performed with the star-shaped sample placed in the focal plane and rastered with a 20 nm step size in a grid of 150 × 150 steps covering a 3 × 3 μm^2^ field of view. The dwell time was 0.25 s per sample position. The incoherent image formed by plotting the total transmission as a function of sample position gives absorption contrast, as shown in Figure 6a. The weak side lobes of the focused beam due to the wavefront roll-off ensured overlap of the illuminated regions of the sample from neighboring points in the scan. This provided adequate redundancy to carry out a ptychography reconstruction of this dataset to give an image with enhanced spatial resolution and contrast. The absorption-contrast coherent image is shown in Figure 6b for comparison with the incoherent STXM image, showing improved signal to noise ratio despite utilizing the same total number of detected photons.

### Manufacturing limits

The quality of an MLL is set by the precision of the deposition process rather than its accuracy, since scale errors simply modify the focal length and operating wavelength. Although the designs of each lens were different, the similarity of the aberrations observed in these lenses suggests that they are caused by layer placement errors that could be avoided by repeating the deposition using a corrected layer thickness recipe, as determined from the measurement. It is reasonable to expect that a better precision can be achieved than that indicated by the difference *φ*_1_(*x*)−*φ*_2_(*y*), equal to approximately 0.2 waves RMS, or by the individual errors of approximately 0.2 waves observed when excluding the edge regions. If extended to 0.0075 NA, such an error should give a Strehl ratio of 0.2 and a resolution approaching that of a perfect lens. Could this precision be maintained for even larger numerical apertures? If the focal length is to be maintained above 1 mm, then one challenge is to grow MLL structures with total thicknesses of approximately 80 μm, as would be needed for 1 nm resolution at 16 keV (0.04 NA) and periods considerably smaller than 1 nm. We have already achieved this thickness using our current deposition infrastructure and demonstrated multilayers with a 0.6 nm period (Supplementary Fig. S2). As periods are reduced, a given layer placement error produces a larger phase error. Non-compensatable aberrations may be characterized by errors Δ*d* in period, which manifest as a phase gradient of the wavefront, ∂*φ*/*∂x*. A fractional error of the period gives rise to a fractional error in the Bragg angle *θ*, and we find that ∂*φ*/∂*x*=(4*π*/*λ*)Δ*θ*=2*π*Δ*d*/*d*^2^ for a period *d.* The phase error accumulated over a period is thus 2*π*Δ*d*/*d*. Random uncorrelated errors in period are tolerable, but any trend (such as occurs in the small-period parts of the lenses reported here) gives rise to significant aberrations. The same reproducibility achieved here with 4-nm period layers would produce wavefront aberrations 7 times larger for a 0.6 nm period. Stability tests of single-period multilayers made before and after long deposition runs for lenses indicate a reproducibility of periods of 0.1% and thus the possibility to produce an improved lens, once aberrations of the first are characterized, in a following deposition run. Slowly varying phase errors, which are those that affect the resolution the most, could be corrected with a phase plate corrector^33^, and chromatic aberrations (due to the large number of layers in the lens) could be reduced by combining the MLL with a refractive corrector to form an achromat^34^.

### Projection imaging

X-ray lenses can be used in a variety of ways to obtain an image. At hard X-ray photon energies, phase contrast imaging has a large dose advantage over absorption contrast, especially for biological samples, which mainly consist of elements of low atomic number elements. In this case, differential phase contrast (using a configured detector)^35^ and ptychography via Wigner distribution deconvolution^36, 37^ are favorable low-dose methods to achieve high-resolution imaging without exceeding tolerable doses of the samples. However, biological imaging below 10 nm resolution may need to rely upon femtosecond X-ray pulses to overcome radiation damage^38, 39^. Such an ‘imaging before destruction’ measurement will only permit a single shot, precluding scanning. We are therefore interested in exploring a full-field phase-sensitive imaging approach for imaging biological samples or fast processes^40^. This could be accomplished by projection imaging, where a magnified hologram is obtained of a sample placed downstream of the beam focus. The resolution in such case is generally limited by the probe size. Although it is possible to account for wavefront errors in the probe, which cause a distortion of the hologram^41, 42, 43, 44^, robust and accurate projection imaging requires an optic with low aberrations.

**Figure 7:**
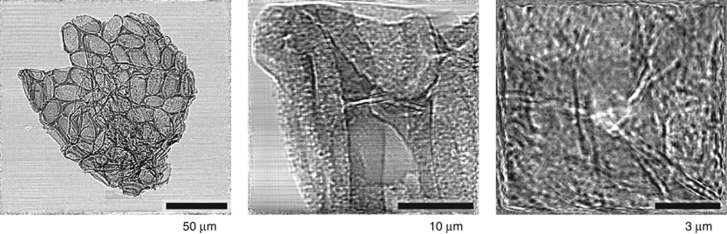
In-line projection holograms of an Acantharian cyst obtained at the PETRA III P11 beamline using 16.3 keV X-rays. The magnification is increased as the sample is moved towards the X-ray focus. The exposure time of the first image was 2 s, over which time 1.7 × 10^9^ photons were recorded. The exposure time of the other images was 5 s, with 3.7 × 10^9^ detected photons.

As a test of the suitability of the MLL pair for single-shot projection imaging, we measured projection holograms of the skeleton of an Acantharian cyst. Acantharia^45^ are marine unicellular planktonic eukaryotes^46^ that use strontium sulfate (celestite) to build a spiny exoskeleton to avoid predation^47^. Some Acantharia form cysts, which are typically round or egg-shaped^48^. Such a cyst provides a useful demonstration of imaging using our MLLs, as it has an intricate structure on length scales from nanometers to hundreds of micrometers. The three projection holograms shown in Figure 7 were obtained with the object placed 11, 1.8 and 0.6 mm downstream of the focus (from left to right), corresponding to demagnified pixel sizes of 430, 70, and 24 nm. A no-sample ‘white field’ intensity map was divided from the measured sample projections to obtain the images shown in Figure 7. No other correction was made. The number of photons recorded in these images was approximately 10^9^ per second. Although not demonstrated here, it is possible to recover an image from the hologram with a resolution dependent on the demagnified pixel size^43^, which could approach 1 nm with a suitable lens and detector.

## Conclusions

A new multilayer material pair, WC/SiC, was used to prepare high efficiency, high-NA MLLs, which were used in a series of studies that indicates their utility for X-ray imaging applications and provides clear indications that creating lenses approaching 1 nm resolution is possible. A high NA requires a large variation in layer period, which posed a challenge for a previous material pair of W/SiC in which the metallic W layers transitioned from amorphous-to-crystalline as the layer thickness increased. The transition resulted in a defect in the wavefront that limited the useable NA. The multilayer system employed here, WC/SiC, consists of carbides that remain amorphous over the entire layer thickness range considered. Lenses with an NA of 0.0075 were fabricated for a photon energy of 16.3 keV. The Rayleigh resolution for such a perfect lens of this NA (with a square pupil) is 4.8 nm. Using ptychography, we characterized the 2D focus to be 8.4 × 6.8 nm at this photon energy. This was larger than the diffraction limit due to a placement error of the thinnest layers in the structure. The form of this error was very similar for both lenses despite their fabrication in separate deposition runs. The effective NA of the lens pair due to this error was 0.006, as validated by incoherent scanning transmission X-ray imaging of a test structure. We measured the efficiency of each lens to be above 80%, which enabled fast acquisition of high-resolution projection holographic images of an Acantharian cyst.

## Figures and Tables

**Table 1:** Parameters of the two lenses used in this study

	Horizontal focusing lens (MLL1)	Vertical focusing lens (MLL2)
Focal length	1.36 mm	2.02 mm
Number of bi-layers	3326	5100
Smallest period	3.90 nm	3.86 nm
Largest period	21.7 nm	15.8 nm
*Smallest 2*θ*	3.5 mrad	4.8 mrad
*Largest 2*θ*	19.5 mrad	19.7 mrad
*NA	0.008	0.0074
Lens height	21.8 μm	29.9 μm
Offset from optic axis	5.0 μm	10.0 μm

The parameters labeled with an asterisk (*) depend on photon energy and are given here for 16.3 keV (0.076 nm wavelength). The deflection angle 2*θ* is twice the Bragg angle given by *λ*=2*d* sin *θ*, where *λ* is the wavelength and *d* the layer period.
