# Supplementary material for: X-ray focusing with efficient high-NA multilayer Laue lenses
Source: Light Sci Appl. 2018 Mar 23;7:17162–. doi: 10.1038/lsa.2017.162 (PMC6060042; doi:10.1038/lsa.2017.162)
Supplement: Supplementary Information [file lsa2017162x1.docx]

**Supplementary Information**

**X-ray focusing with efficient high-NA multilayer Laue lenses**

Saša Bajt^1^, Mauro Prasciolu^1^, Holger Fleckenstein^2^, Martin Domaracký^2^, Henry N. Chapman^2,3,4^, Andrew J. Morgan^2^, Oleksandr Yefanov^2^, Marc Messerschmidt^5^, Yang Du^2^, Kevin T. Murray^1^, Valerio Mariani^2^, Manuela Kuhn^1^, Steven Aplin^2^, Kanupriya Pande^2^, Pablo Villanueva-Perez^2^, Karolina Stachnik^1^, Joe P. J. Chen^6^, Andrzej Andrejczuk^7^, Alke Meents^2^, Anja Burkhardt^1^, David Pennicard^1^, Xiaojing Huang^8^, Hanfei Yan^8^, Evgeny Nazaretski^8^, Yong S. Chu^8^, Christian E. Hamm^9^

^1^Photon Science, DESY, Notkestrasse 85, 22607 Hamburg, Germany

^2^Center for Free-Electron Laser Science, DESY, Notkestrasse 85, 22607 Hamburg, Germany

^3^Department of Physics, University of Hamburg, Luruper Chaussee 149, 22607 Hamburg, Germany

^4^Centre for Ultrafast Imaging, Luruper Chaussee 149, 22607 Hamburg, Germany

^5^National Science Foundation BioXFEL Science and Technology Center, 700 Ellicott Street, Buffalo, NY 14203, USA

^6^Department of Physics, Arizona State University, Tempe, AZ 85287, USA

^7^Faculty of Physics, University of Bialystok, Ciolkowskiego 1L Str., 15-245 Bialystok, Poland

^8^National Synchrotron Light Source II, Brookhaven National Laboratory, Upton, NY 11973 USA

^9^Alfred-Wegener Institute, Helmholtz Center for Polar and Marine Research, Bussestr. 27,

27570 Bremerhaven, Germany

Correspondence: Saša Bajt, E-mail: [sasa.bajt@desy.de](mailto:sasa.bajt@desy.de)


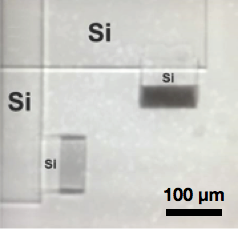


**Supplementary Figure S1:** Screenshot of two MLLs, one already tilted to Bragg condition (black, top right) and one that has not been aligned yet (more transparent, bottom left).

**Supplementary Figure S2:** Small angle X-ray diffraction measurement of a periodic WC/SiC with a period of 0.6 nm showing the first Bragg peak at *θ* of about 7.5°.
